# Research Advance Directives: Ethical Implications for Persons with Alzheimer’s Disease, and for the Families of Elderly Dementia Patients

**DOI:** 10.1017/jme.2025.10185

**Published:** 2025

**Authors:** Dean Evan Hart

**Affiliations:** Global Public Health, NYU, United States

**Keywords:** Alzheimer’s, Palliative Care, Dementia, Advance Directives, Neurodegeneration

## Abstract

As both human longevity and diagnostic ability improve, more individuals are being diagnosed with Alzheimer’s dementia disease (Alzheimer’s). Yet there is a paucity of new Alzheimer’s research trials. One obstacle to research is the large number of Alzheimer’s patients deemed incapable of providing informed consent for clinical research. Research advance directives (RADs) offer patients the opportunity to provide informed consent before incapacity occurs. However, critics question whether RADs guarantee informed consent, claiming that due to the nature of the disease, the consenting agent is no longer the same person after becoming incapacitated. This paper assesses the debate while using a conception of personhood, informed by the latest Alzheimer’s research, which does not reduce the concept of personhood to psychological capacities. It explains how personal identity can persist despite Alzheimer’s, such that RADs can and should suffice for informed consent.

## 1. Introduction

In patients with Alzheimer’s dementia disease (Alzheimer’s), there is an irreversible, presently untreatable degradation of memory and personhood as a result of progressive brain lesions. Advance directives (ADs) are documents that set out Alzheimer’s patients’ wishes regarding, but not limited to, their care and treatment after they lose their autonomy and ability to provide informed consent. ADs are vital, as they represent an individual’s most personal beliefs and essential principles. They capture, in writing, how patients envision their end-of-life care. To achieve this goal, patients rely on factors such as their personal history, life experiences, religion, culture, and values. Most importantly, ADs allow, and indeed require, that individuals have their wishes respected even when they can no longer communicate those wishes. The federal authorization of ADs through the Patient Self-Determination Act (1990) provides a legal basis for the implementation of ADs by mandating that patients be informed about ADs and provided with the option of creating them before losing the ability to do so.^1^

There is an imperative to further the research agenda for Alzheimer’s, as this provides the possibility of finding a cure, effective treatments, and/or promoting our understanding of the nature of the disease. However, the nature of the disease itself complicates this endeavor in a way that does not apply to other serious diseases, such as cardiovascular disease or cancer. Informed patient consent is a necessity for participation in all medical research trials. Because of this requirement, once Alzheimer’s patients are no longer able to provide informed consent due to neurodegeneration, they are excluded or removed from participating in such trials.^2^

Research ADs (RADs) thus present an opportunity to overcome this problem through what Ronald Dworkin calls *precedent autonomy*: the will of a competent individual can be recorded and expressed even after the loss of the mental capacities typically associated with such autonomy.^3^ This requires patient understanding, self-determination, and the ability to take risks voluntarily and be bound to benefit others in future generations when there is no reasonable likelihood that the medical protocol will be of benefit to one’s existence. Patients can provide consent in advance for participation in future research that might occur after they can no longer provide such consent. RADs express permission to detail what specific research, if any, is permitted by Alzheimer’s patients**,** and whether there are any limitations to such research as applied to a particular Alzheimer’s patient.

Despite these benefits, RADs face a powerful objection from Rebecca Dresser’s “changing selves” argument.^4^ Dresser argues that the neurodegenerative effects of Alzheimer’s led to a rupture and erosion of the psychological continuity and mental capacities that are usually understood as grounding personal identity. The consequence of this is that, according to Dresser, the person who writes a RAD is not the same person as the one who is subjected to the RAD. Because of this, the person who writes the RAD has no right to decide on the medical treatment of the person who is subjected to the RAD. Dresser’s argument significantly undermines Dworkin’s idea of precedent autonomy, undermining the bioethical basis of ADs.

This paper argues against Dresser’s claim that Alzheimer’s entails a change in personal identity by drawing on recent empirical research and theoretical understandings of personhood that expand that concept beyond mental capacity. Suppose we are to understand personhood, which accommodates the changes individuals undergo in Alzheimer’s dementia. In that case, we must have an expansive understanding of what makes a person a person, i.e., one that is not dependent on mental capacities such as memory recall. If we focus on what Dworkin calls the “whole person,” a view that encompasses underappreciated facets such as legacy, aesthetics, taste, appearance, social etiquette, caring, dancing, music, and gestural communication, then we are able to see how personhood persists despite the neurodegenerative effects of Alzheimer’s.

## 2. Discussion

### 2.1. Autonomy and Personhood in Medical Ethics

*Autonomy*, or freedom from external constraints and internal limitations, is a fundamental concept in medical and research ethics. An external constraint occurs when others prevent or interfere with an individual making free decisions — for example when one is imprisoned or kept ignorant of vital information. An internal limitation relates to the agent’s own decision-making capacities — for example, impairment due to brain underdevelopment, injury, or degeneration. An example of an internal limitation would be children’s limited neurological development, which implies that they have less decision-making capacity than that of mature, healthy adults.

*Personhood* is essential to autonomy, as it allows and permits the capacity for self-determination that defines autonomy. Personhood as a concept has a long and persuasive history. A classical statement of what makes a person a person comes from philosopher John Locke, who claimed that a person is “a thinking intelligent being, that has reason and reflection and can consider itself as itself, the same thinking thing, in different times and places”.^5^ This statement elucidates the fundamental mental properties traditionally associated with personhood: self-consciousness, rationality, and persistence over time. If one is self-aware, possesses reason, has the capacity to make judgments, and uses language, all of which abilities persist over time, then one is considered to be a person. As we will see below, there have been many criticisms of this conception of personhood. But what is essential for our purposes here is that autonomy requires a person to have the capacity to think for oneself and to have a stable awareness of one’s nature and characteristics over time.

Respect for autonomy is critical to medical ethics. Treatment, care, and research cannot be properly imposed on unwilling or encumbered individuals. This idea manifests in the concept of *consent.* Anything done to an agent (the patient) requires their approval. Therefore, agents (patients) must agree to and provide permission for the treatment or research in which they will participate.

However, mere consent — that is, the act of providing permission — is insufficient for the standards of medical ethics. Agents must also understand exactly what they are consenting to: the nature of the procedures involved, their risks, benefits, and possible alternatives. Lack of information can be considered to be an external constraint, as agents cannot act autonomously if they are not aware of all of the relevant considerations needed to make an informed judgment. Healthcare providers must obtain *informed consent*; that is, consent provided by a capacitated agent who knows all of the risks, benefits, and alternatives of a given procedure.^6^

Not all constraints on informed consent are external, however. Legally, individuals can only provide informed consent if they are deemed competent.^7^ Competence itself is defined by the possession of sound mental capacities. The relevant capacities vary to some extent, but involve the capacities to reason and deliberate, hold appropriate values and goals, appreciate one’s circumstances, understand information given, and communicate choices.^8^ These capacities must be present as continuous qualities over time.

In a clinical setting, there are various frameworks for assessing capacity. These are the MacArthur Competence Assessment Tool-Treatment (MacCAT-T), Appelbaum’s schema or, in the UK, the schema set out by the Mental Capacity Act (2005).^9^ Although some insist on a strict delineation between capacity and competence, with the former being a medical term related to treatment decision-making and the latter being a broader legal concept, Appelbaum uses the terms interchangeably because “the oft-cited distinctions between them … are not consistently reflected in either legal or medical usage”.^10^ These tools draw on the legal definition of competence to distinguish four areas of capacity: expression of a choice, understanding the information that is presented regarding the proposed treatment, appreciation of the situation as it pertains to the patient, and rational manipulation of information.^11^

Informed consent is a requirement for all scientific research trials, except under certain unusual circumstances.^12^ These circumstances fall into three different categories: (a) when obtaining consent is unduly burdensome, (b) when the proposed research does not violate patient autonomy, and (c) when there are important public health and clinical aims being fulfilled by the research. The US Department of Health and Human Services has a set of mandatory conditions that must be met if consent is to be waived in a research study.

The ethics of informed consent often focus on the external barriers to informed consent for fully capacitated individuals: i.e., epistemic and practical barriers impeding individuals from grasping the full range of information they should possess before providing consent. The ethics of informed consent in the context of Alzheimer’s shifts the focus to the possibility or impossibility of individuals being able to provide informed consent while undergoing progressive neurological deterioration. As we will now explain, the progression of Alzheimer’s coincides with the gradual loss of the mental capacities that are seen as constituting legal competence or, in bioethics, the loss of personhood and autonomy. These conditions, however, often do not apply to research trials involving individuals with Alzheimer’s.

This situation raises complex ethical questions, such as: how can we respect the autonomy of Alzheimer’s patients while addressing their inability to provide informed consent for research that could benefit them as well as future generations? Because of this, we must consider the difficulties that arise when an Alzheimer’s patient might be unable to provide informed consent for a research trial that requires it.

### 2.2. The Threats to Autonomy from Alzheimer’s Disease

Alzheimer’s is defined by the progressive development of aberrant “misfolding” proteins, which lead to the loss of the mind’s function and brain homeostasis. Alzheimer’s causes 60% to 70% of all dementia cases. It leads to the gradual loss of cognitive health and competence and, thus, to a lack of functional independence. The most significant symptoms of Alzheimer’s are memory loss, problems with language, disorientation, as well as behavioral and mood-related issues. As the disease progresses, bodily functions are also lost, leading to total dependency, and ultimately, death. There are no known cures for Alzheimer’s. The only known palliative treatments alleviate some symptoms but can neither stop nor reverse the disease’s progression.

By degrading cognitive and functional capacities, Alzheimer’s causes the gradual loss of autonomy and, thus, loss of the ability to give informed consent to various medical procedures due to irreversible brain cell dysfunction and death, and is a terminal diagnosis. In late-stage Alzheimer’s, when individuals can no longer move or speak, the lack of autonomy is clear-cut. What is unclear is the degree of remaining autonomy of individuals with mild-to-moderate Alzheimer’s, since they may be capable of articulating preferences despite significantly impaired decision-making ability.^13^ As identified by Kim, one study found that 40% of participants with mild cognitive impairment were judged incapable of providing informed consent, whereas another study found that 62% of individuals with mild to moderate Alzheimer’s did not meet the threshold on at least one standard of capacity for research consent.^14^ As Alzheimer’s degrades an individual’s autonomy and sense of self, the capacity to provide informed consent is thus also degraded, but the degree of degradation seems to vary from study to study and is poorly predicted.

While in some cases, individuals can participate in research trials even if they are unable to provide informed consent, which is likely to require a surrogate’s approval, lack of informed consent is generally a significant barrier to research study participation. Many such research trials mainly focus on upstream barriers once an individual has been found eligible — on issues such as funding, transportation, and willingness to participate.^15^ The greatest barrier, however, is likely to be downstream as a result of the stringent eligibility criteria that bar individuals with cognitive impairment.^16^ In one study, for example, 3,290 individuals were screened for a risk-reduction factor for an Alzheimer’s research trial. Out of all these individuals, only 28% were found eligible to provide consent, thereby excluding the vast majority of potential participants.^17^ A meta-study of Alzheimer’s research trials found that preclinical trials have an average screen failure rate of 88%, which researchers attribute to “specific cutoffs for neurocognitive status.”^18^ Such exclusions severely restrict the diversity and generalization of research findings. Given the pressing need to increase participation in Alzheimer’s research trials, this practice of disqualifying out of hand most potential participants as a result of concerns over informed consent should be reviewed and reconsidered urgently.

### 2.3. RADs as the Solution for Patient Exclusion

One solution to the issue of patient exclusion is the use of RADs, an extension of ADs. These are legal documents that record an individual’s preferences regarding significant medical decisions — procedures an individual desires or rejects — in the event that they are unable to articulate these preferences contemporaneously due to incapacity.^19^ ADs gained institutional support in the United States after the federal Patient Self-Determination Act was enacted. This Act made it a legal requirement for medical facilities that receive federal funding to inform incoming patients about their rights in deciding on their medical treatment. Many ADs also appoint a surrogate or proxy, who is supposed to act as a facilitator and custodian of the individual’s desires regarding medical care by making decisions in accordance with the patient’s wishes.

ADs are used by individuals with Alzheimer’s to set out how they would like their end-of-life care to be provided, once they become mentally incapacitated and thus are incapable of making such decisions. While ADs usually relate to major medical decisions, such as whether one would like to be intubated or receive CPR, they may also include religious or ethical preferences, such as whether one retains a kosher or vegetarian diet despite Alzheimer’s. What is important about advance directives is that they enable autonomy for incapacitated persons through what Dworkin called *precedent autonomy.* This is the idea that autonomy can often be established through precedence: the will of a competent individual can be recorded and expressed even after the loss of the mental capacities typically associated with autonomy.^20^

ADs can be made by any legally competent adult; however, they are frequently made by individuals with Alzheimer’s or individuals at risk of the disease. For the former, this occurs in a time frame between diagnosis and before significant cognitive dysfunction is such that they are deemed legally incompetent. Individuals can complete ADs with mild cognitive impairment present, but they must still be deemed competent to do so. In ideal conditions, most individuals at risk of Alzheimer’s disease would have completed and maintained an AD before symptoms, signs, and diagnosis occurred. The timeframe in which an AD ought to be completed remains a contentious issue.^21^

RADs utilize the AD framework in the context of research instead of treatment. Instead of taking a stance on major medical procedures, individuals set out their preferences regarding research involvement.^22^ The provisions of a RAD can include participation in clinical trials as well as the donation of one’s brain after death. Thus, individuals can also declare themselves as willing research participants prior to the loss of their capacity to provide informed consent.^23^ This allows them to realize their values and, most importantly, to express personal beliefs and legacy by aiding scientific research and helping to prevent others in the future from experiencing the worst effects of Alzheimer’s.^24^ RADs thereby present a valid solution to the problem identified in the prior section: if informed consent is provided by a competent individual and documented in an AD, then this will allow individuals to participate in research trials even after Alzheimer’s renders further informed consent no longer possible. The use of RADs, therefore, would be likely to increase the number of eligible participants for Alzheimer’s research trials, thus allowing and encouraging an acceleration of Alzheimer’s research.

One immediate objection to the use of ADs in general is the possibility of redetermination, i.e. a potential divergence between what is set out in the AD and what an individual with Alzheimer’s might want at some later time. What this issue points to is the differences that might develop between the *person of a lifetime* — the capacitated individual who writes the AD — and the *person of the moment* — the incapacitated individual who will be subject to the AD.^25^ The primary example used by critics is that of an individual with moderate to severe cognitive impairment who might be said to enjoy their day-to-day life, and would like it to continue if possible, but who has an AD with a DNR order. In this case, critics argue that the use of an AD by the person of a lifetime violates the person of the moment’s right to life. While this argument will be addressed in depth in latter sections, it will help to address it briefly now in order to demonstrate a key difference between ADs with RADs, as well as clarifying the framework for which RADs are being advocated.

ADs can be understood as either *sovereign* or *supplemental.* If an AD is sovereign, then it is the authoritative record of the preferences and wishes of an individual, such that the person of a lifetime supplants the person of the moment if the two come into contradiction. If an AD is supplemental, however, it is meant to supplement the wishes of the person of the moment, such that the person of a lifetime provides further authority or credence to the preferences of the person of the moment. A vital difference between ADs regarding medical treatment and RADs is that the former operate in a context in which they must be sovereign, as they relate to procedures that an individual necessarily can no longer decide on: do-not-resuscitate orders or directives regarding intubation are relevant at the time in which an individual is totally incapacitated physically or mentally and therefore can no longer opine on what further medical treatments they want. In contrast, RADs operate in a context in which individuals have reduced capacity but may still be able to communicate preferences on some level or in some manner. They are meant to supplement the preferences of an individual who would otherwise be regarded as incapable of providing informed consent but nonetheless wish to participate in research trials.

A RAD is not a contract binding an individual to participate in research trials; rather, it is a document that sets out an individual’s future wishes. Its use should thus allow willing individuals to participate in later research trials even after there may be concerns regarding their ability to provide informed consent: RADs’ purpose is not to bind individuals to any set of actions but rather to prevent them from being barred from willingly participating in research trials. They should therefore never be used to compel unwilling patients to participate in research. Thus, unlike ADs regarding medical procedures, RADs operate in a supplemental rather than sovereign manner: they represent the agreement of the person of a lifetime and the person of the moment.

Moreover, there are strong reasons to believe that RADs resolve issues present in current research practices while also shoring up other practices. While patient exclusion is itself an issue, the means by which patient exclusion occurs is also problematic, i.e. the judgment by a medical professional that an individual is incapable of providing informed consent. While the means of assessing mental capacity vary, they all depend on the judgment of a clinician. The issue with this is that studies often show high rates of variance in the assessment of capacity by clinicians. One study had five clinicians evaluate the capacity of patients with Alzheimer’s; they only agreed in 56% of cases.^26^ Another study had 99 psychiatrists assess whether an individual patient could provide informed consent. This resulted in a 40-60 split judgment, with the former finding the individual capable and the latter finding the patient incapable.^27^ In one of the most exhaustive studies performed, five psychiatrists evaluated the 555 capacity interviews of 188 patients, resulting in “wide variation in judgments of capacity.”^28^ While the majority of opinions from panels made up of experts are more reliable, their resource intensity makes them impractical.^29^ Although there are studies that demonstrate acceptable levels of agreement among experts, the number of studies demonstrating otherwise points to an unacceptable level of variability in clinical judgments of capacity.^30^ This level of variability goes beyond normal human error, at times coming close to a coin flip, and should raise major doubts regarding the practice of making clinical judgment of capacity the final arbiter of whether patients can be allowed to participate in research trials. This variability gives further reason to allow individuals to use RADs to preemptively provide informed consent for participation in research trials.

Another issue that RADs help resolve has to do with surrogates or proxies. In the case of Alzheimer’s, mental incapacitation leads to a stakeholder, most often a family member, becoming the healthcare proxy and designated surrogate. This individual takes on the ultimate responsibility for decision-making on behalf of the patient and power of attorney. The alleged motivation behind this practice is usually that some stakeholders, through their expertise or intimate knowledge of the patient, can best act faithfully on behalf of the incapacitated Alzheimer’s patient.

However, there are conflicting accounts of what should motivate a surrogate. The classical model of surrogacy was explicitly paternalistic: many stakeholders were allowed to decide on what would be in the patient’s best interest. This interest was defined independently of all of that patient’s previous wishes. Today, surrogates are more often seen as taking on an empathetic role, in which they use their alleged intimate knowledge of the individual with Alzheimer’s — their understanding of his or her legacy — to make decisions which they believe the patient would make if given the capacity to do so; this is known as the substituted judgment model.

In some research trials, if an individual with Alzheimer’s was found unable to provide informed consent, decisions about participation fell to the surrogate. However, this depends on the ability of surrogates to accurately predict and then express the wishes of the individual with Alzheimer’s. Various issues and complications arise from this attempted conveyance of responsibility. Surrogates hold a wide range of views regarding their roles and what they are meant to do, which means that many surrogates disagree with the substituted judgment model.^31^ Even if some surrogates do commit to the substituted judgment model, various studies have shown that they are unable to predict patient preferences accurately.^32^ In one such study, even though respondents overwhelmingly believed that their physicians and family members would accurately represent their wishes, neither group was able to do so.^33^ In fact, surrogates are often found to consistently project their own desires and values onto the individuals whom they are meant to represent.^34^ In a study conducted by Marks & Arkes, the judgments of 62.5% of surrogates were found to represent their own wishes for the patient rather than the patient’s actual preferences.^35^ In a systematic review of the literature, surrogates incorrectly predicted patients’ treatment preferences in one-third of cases.^36^ This failure led the physicians who conducted this review to recommend other alternative mechanisms to predict patients’ end-of-life treatment preferences more accurately. An AD may contain a living will providing moral authority for the end-of-life treatment or lack thereof when voiceless.

Given the significant problems with the reliability of clinical assessments of capacity and surrogate approximations of a patient’s wishes, RADs represent an important and persuasive means of allowing individuals with Alzheimer’s to participate in research trials. They, of course, do not replace valid alternatives, nor do RADs work against them. In fact, a further benefit of RADs is in relation to the heavy burden of responsibility and associated moral distress that is placed on clinicians and surrogates when having to decide on patient involvement without any input from the patients themselves.

### 2.4. The Changing Selves Argument Against RADs

The preceding section provides policymakers with strong reasons to strengthen and widen the usage of RADs for individuals with Alzheimer’s. Nonetheless, debates remain about the usage of RADs from bioethical, clinical and legal perspectives.^37^ These debates, however, tend to relate to particular aspects of RADs or to concerns relating to their implementation. This paper is primarily concerned with responding to what it sees as, on a bioethical level, the most substantive objection to RADs. This objection relates to the fundamental moral authority of ADs on a conceptual level, and, as such, presents a fundamental problem. This concern can be summarized in the following question: *Who is the person providing the consent?*

ADs assume that the person who provides informed consent for participation in research trials is the *same* person who will then go on to participate in such research trials. Individuals with Alzheimer’s, however, undergo what some researchers call a “cognitive transformation.”^38^ This transformation — the gradual loss of core mental capacities and, in particular, memory recall — leads some to question whether the person who writes the RAD is in fact the same as the person who will be subjected to it.

This criticism has its grounds in the classical conception of personhood discussed above, which requires persistence of self and rational capacities, both of which therefore are, in these accounts, considered necessary components of personhood. A more significant contemporary influence on this criticism is Derek Parfit’s “reductionist view” about personal identity.^39^ On this account, what establishes identity between persons — i.e., whether a person at one period is the same person at a later period — is a form of psychological continuity dependent on mental capacity, particularly memory recall. The result of this claim is that a traumatic brain injury, or in our case progressive neurodegeneration, can cause a change in personal identity: that is to say, it can cause one to no longer be the same person. Parfit also uses the language of “selves” and believes that his arguments give us reason to think of ourselves as having “successive selves” in a single lifetime, meaning that disruptions and changes in psychological continuity lead us to be more than one person in a single lifetime.^40^ This claim can lead to two different conclusions, both of which may appear to undermine the authority of RADs.

One possible conclusion is that Alzheimer’s causes the cessation of personhood. The Lockean standard holds faculties such as memory recall and rationality to be necessary conditions for personhood. Therefore, their loss due to neurodegeneration allegedly makes late-stage Alzheimer’s patients cease to be “persons” or to become “non-persons.” The implication is that the person who created the RAD no longer exists and, therefore, cannot continue to provide consent. According to this argument, since non-persons cannot consent to RADs, lacking competence, then any individual whose Alzheimer’s condition has sufficiently progressed to such a late stage cannot be allowed to participate in research trials. We must, however, note that few clinicians nowadays endorse this view, once associated with Peter Singer, that individuals with Alzheimer’s are non-persons, although it persists among some involved in Alzheimer’s care.^41^

Rather, now the focus has moved from personhood to personal identity: instead of asking whether individuals with Alzheimer’s are indeed persons, both researchers and philosophers now focus on whether an individual with Alzheimer’s meets the continuity requirements of personhood. This second objection holds that the person who signs the RAD is no longer the *same* person once they are seriously impaired by Alzheimer’s.^42^ This argument has been developed by Rebecca Dresser in order to argue against the validity of ADs, and a similar argument has been developed by Dominic Wilkinson.^43^ Dresser’s “changing selves” argument is simple yet appears to be effective.^44^ It can be summarized as follows:

1. The validity of an AD in the context of Alzheimer’s is dependent on continuous identity between the person who creates the AD and the person who will be subject to the AD.
2. Personal identity is grounded in psychological continuity and the persistence of mental capacity.
3. The neurodegenerative effects of Alzheimer’s disease cause a rupture of psychological continuity and erosion of mental capacity.
4. (1-3) Therefore, Alzheimer’s disease causes a rupture in personal identity between the person who writes an AD and the person who is subject to the AD.
5. (1-4) Therefore, an AD is not valid in the context of Alzheimer’s.

By “validity” I refer to the moral authority or that which justifies the usage of an AD. The person who writes an AD is taken as having the right to do so, i.e. to decide on the medical treatment or research involvement of the future self, based on the assumption that they are deciding on treatment for themselves. However, the contention of Dresser and Wilkinson is that the situation is more analogous to one person deciding on the treatment of another person, and this supposedly justifies what Wilkinson calls “identity relative paternalism”: we ought to prevent anyone from doing to their future selves what it would be wrong to do to other people, because a future self is, in effect, another person.^45^

Thus, in Wilkinson´s view, the person who writes an AD does not have the right to decide on the medical treatment of the person who will be subject to the AD; rather, such treatment should be determined by medical professionals with a view to the well-being of the person of the moment. It is important to note that Dresser first developed her argument explicitly in opposition to Dworkin’s concept of precedent autonomy, and therefore it should be understood as taking direct aim at the bioethical basis of ADs. As well as applying to ADs, Dresser’s argument applies to RADs, which she has also argued against.^46^ If one follows her argument, the person who wrote the RAD is not the same person as the individual who will participate in the research trial, and thus that earlier person cannot provide informed consent for the latter. If these arguments are correct, then RADs would suffer significant ethical problems, ones which would make practical clinical or legal concerns not only extraneous, but indeed irrelevant.

I have previously responded to Dresser’s view through scrutinizing the first premise of her argument, arguing that, in fact, a reductionist account of personal identity ought to lead one to reject the view that the moral authority of ADs is dependent on strict identity between the AD author and the AD subject. This demonstrates that Dresser’s argument is invalid even if one grants the premise that there is a difference in identity between the maker of an AD and the subject of an AD. In this paper, however, I argue against the claim that the neurodegenerative disease effects of Alzheimer’s lead to a rupture in personal identity, or at least in any way that invalidates the use of ADs. A concern with the dignity and autonomy of individuals with Alzheimer’s should lead us to understand personhood as being fully beyond the confines of mental capacity and psychological identity.

### 2.5. Personhood Beyond Mere Mental Capacity

The classical conception of personhood, as discussed in section 2.1, is one that is intrinsically based upon consciousness and rationality. Personhood, in this format, is reducible to a set of mental capacities.^47^ In the latter half of the 20th century, this idea was criticized by philosophers and social scientists who focused on factors such as gender, race, and disability.^48^ The classical conception of personhood, they argued, was an abstract and idealized notion incommensurate with the vicissitudes and diversity of human life. Given the role that personhood plays in ethical, political, and legal discourse, it makes eminent sense that we ought to revise or even eliminate the concept if it is incapable of meeting all of the criteria required of the concept and implied by it.

The meaning of personhood among individuals with Alzheimer’s is a prime example of this issue. While some researchers were ready to deny the personhood of individuals whose advanced Alzheimer’s had progressed past a certain point, or at least to argue that they were not the same person as they had been before the onset of the disease, others like Ronald Dworkin took this problem as a sign that the meaning of personhood must be reconceptualized.

While Dworkin did not reject the idea that rationality played a key role in personhood, he also argued that the difficulties involved in tackling end-of-life care for individuals with Alzheimer’s require us to consider the “whole person.”^49^ Instead of reducing personhood to a mere mental capacity, personhood should be defined by *total legacy* — that is, the patient’s values, tastes, life experiences, projects, character traits, and relationships. These elements provide “genuine meaning and coherence to our lives” and represent expressions of our true selves.^50^ This approach establishes a reciprocal relationship between autonomy and personhood, as our personhood comes to be defined by our exercise of autonomy: the beliefs, actions, and judgments which we choose to express and realize culturally in our lives.

The idea of legacy as an inherent, constituent part of personhood can be traced back to Aristotle, who claimed it was possible to wrong even the dead.^51^ Aristotle’s holistic integrated approach enabled him to give persons their due even once they were no longer alive. Analogously, then, focusing on the legacy of a whole person can enable us to give Alzheimer´s patients their rightful expectations even when the progression of brain cell dysfunction and cellular death of Alzheimer’s has undermined many of the mental capacities usually associated with personhood.

These alternative arguments, however, depend on a reductive conception of personhood, which is at odds with contemporary empirical research on Alzheimer’s disease and on personhood itself. In arguing that individuals with moderate- to late-stage Alzheimer’s are non-persons or different persons from those whom they were before the disease’s progression, neither Singer nor Dresser cite any research — qualitative or quantitative — on personal identity and Alzheimer’s. Instead, they operate solely on the assumptions provided by the outdated classical conception of personhood. Implicit in this outdated view is a mind-body dualism that privileges the former while consigning the latter to a passive or non-existent role.^52^ According to this view, personhood is found only in the mind, whereas the body is merely something that the mind uses. It is through consigning personhood only to the mind and then reducing the mind to a reductive set of mental capacities that the claim that Alzheimer’s entails the loss or rupture of personhood is able to be made.^53^ This conception of personhood has often been deemed deficient, and even detrimental to understanding, treating, and managing Alzheimer’s patients.^54^

Diverse interdisciplinary research from phenomenologists, psychologists, and cognitive scientists has undermined this outdated view, as cognition and, thus, personhood are increasingly understood as being broadly embodied and situated phenomena. Rather than having a simplistic one-way relationship, the mind and body operate through a symbiotic relationship in order to enable cognition. As a result, personhood becomes grounded in body-memory: i.e., “the embodiment and enactment of familiar habits, practices and preferences.”^55^ While embodied cognition is a broad approach and is not without its critics, this reconceptualization — of personhood as being embodied, instead of treating personhood as a mere psychological capacity — is important for our purposes. It renders intelligible the ways in which personal identity can persist in individuals with Alzheimer’s, even after they lose linguistic and/or other rational capacities.

Research from the last two decades demonstrates a greater persistence of personal identity throughout the progression of Alzheimer’s disease than was previously thought to be the case. This research applies to the particular capacities associated with personhood, as well as to expanded conceptions of personhood. In one study, the ability for linguistic and visual self-recognition was investigated among seventy-eight adults with a range of cognitive impairments as a result of Alzheimer’s.^56^ In terms of linguistic self-recognition, and although language usage decreased with cognitive impairment, there were no significant differences in the rates or proportions of pronoun and attribute usage. Visually, individuals could identify themselves in photographs, even if they had forgotten the photographic session which had occurred only minutes before. Another result fully demonstrated that individuals with Alzheimer’s who were unable to overtly or declaratively recognize themselves were still able to recognize themselves unconsciously.^57^ Systematic studies on awareness and insight in individuals with Alzheimer’s demonstrated that the persistence of both capacities was underestimated.^58^

In terms of embodiment, there is a wealth of literature that points to the use of music as a vital tool for reaffirming personal identity in individuals with Alzheimer’s.^59^ Patients demonstrate strong positive emotive responses to music which they enjoyed when they were younger, even in cases of late-stage dementia where verbal communication has ceased. This demonstrates a capacity for bodily memory even when the usual forms of active or verbal recall have ceased.

Whereas Dresser might claim that radical changes in taste suggest a change in personal identity, research has demonstrated that individuals with Alzheimer’s do in fact undergo significant impairment of gustatory function without losing their sense of identity.^60^ Pia Kontos conducted an eight-month ethnographic study of a group of thirteen individuals with Alzheimer’s in a long-term care facility in Canada with conditions ranging from moderate to severe cognitive impairment.^61^ She found that most participants, to varying degrees, retained their characteristic forms of self-expression through appearance, social etiquette, caring, dancing, and gestural communication. For example, one participant, Molly, was severely cognitively impaired, incontinent, and decrepit, yet she always ensured that she wore and displayed her characteristic pearl necklace. These studies strongly support the modern view that the “whole person” subsists through forms of bodily memory even as the mind slips away.

Finally, significant research demonstrates that individuals with Alzheimer’s retain their own sense of legacy. Many studies have utilized Harré’s social constructionist theory of selfhood. This theory divides the self into three types: the first self is characterized by the embodied sense of being a person, the second self is characterized by being a persisting individual with a life history and personal attributes, and the third self is characterized as the social self, i.e. the identity that is demonstrated in relations with others. This second self represents what we previously understood as a person’s legacy. Three different studies conducted using this paradigm found that, although this sense of self undergoes changes from terminal incurable dementia, participants would continue to perceive themselves as being the same person whom they were before the onset of Alzheimer’s.^62^

Collectively, the research surveyed here provides sufficiently strong reasons to reject Dresser´s and Singer’s claims that personhood is either ruptured or lost as a result of the onset and continuation of Alzheimer’s.^63^ This is not to claim that parts of personhood or self-identity are not lost or harmed through the progression of Alzheimer’s, nor to provide a conclusive answer about personal identity loss in Alzheimer’s cases. Instead, I have sought to approach the question of personal identity in Alzheimer’s through an empirically grounded methodology rather than through philosophical preconceptions and assumptions. If contemporary research and our obligation to treat Alzheimer’s patients with dignity both require breaking with the classical conception of personhood, in the elderly neurodegeneration process this result is more than justified by the importance of making such a valid and accurate determination.

It is clear, then, that the argument against the use of RADs due to alleged personal identity issues would demand considerable further support and explanation before it can be deemed to be a real ethical concern that should impede policymaking or cast any doubt on the validity of ADs and RADs. Rather, it is clear that we do not currently possess any substantive reasons to believe that the individual who makes a RAD is necessarily distinct from the person who will be subject to it. Therefore, patients’ informed consent, as expressed in valid RADs, should presumptively and legally determine Alzheimer’s patients’ wishes, which must then be followed by caregivers.

## 3. Conclusion

Too many individuals with Alzheimer’s are excluded from research trials because of concerns surrounding their informed consent. In this paper, I have argued for revising and updating our understanding of personhood and consent to better do justice by respecting the expressed wishes of both present and future Alzheimer’s patients. While in the past, individuals with Alzheimer’s might have been wrongfully considered to be non-persons or different persons from whom they were before the advent of Alzheimer’s, contemporary research and expanded conceptions of personhood allow us to see how patient personhood persists and continues despite cognitive impairment. This reconceptualization enables us to understand how precedent autonomy works through the use of RADs — how we can thus enable the voiceless to have a voice.

This allows us to avoid the concern that the person of the lifetime is not the person of the moment. In avoiding this, the most substantive bioethical objection against RADs is deflated. The person who has the capacity to provide informed consent, the person who makes a RAD, and the person who participates in research trials are all the same person. This does not mean, of course, that no concerns can be held regarding the legal or clinical practicalities of RADs, or of other bioethical issues related to them. However, it does ensure that the moral core of RADs remains intact.

If patients provide informed consent in a RAD to participate in research trials and still wish to do so despite reduced mental capacity, then they should be allowed to do so. The autonomy of the patient should be the guiding principle. In this case, precedent autonomy bolsters an otherwise diminished mental capacity. RADs represent an ethical alternative to excluding patients with Alzheimer’s from research trials, a practice that is holding back both autonomy and science.
